# Application of SARS-CoV-2 Serology to Address Public Health Priorities

**DOI:** 10.3389/fpubh.2021.744535

**Published:** 2021-11-23

**Authors:** Amy C. Sherman, Teresa Smith, Yerun Zhu, Kaitlin Taibl, Jessica Howard-Anderson, Taylor Landay, Nora Pisanic, Jennifer Kleinhenz, Trevor W. Simon, Daniel Espinoza, Neena Edupuganti, Skyler Hammond, Nadine Rouphael, Huifeng Shen, Jessica K. Fairley, Srilatha Edupuganti, Jaime A. Cardona-Ospina, Alfonso J. Rodriguez-Morales, Lakshmanane Premkumar, Jens Wrammert, Rick Tarleton, Scott Fridkin, Christopher D. Heaney, Erin M. Scherer, Matthew H. Collins

**Affiliations:** ^1^Division of Infectious Diseases, The Hope Clinic of the Emory Vaccine Center, Emory University, Atlanta, GA, United States; ^2^Division of Infectious Diseases, Brigham and Women's Hospital, Boston, MA, United States; ^3^Rollins School of Public Health, Emory University, Atlanta, GA, United States; ^4^Division of Infectious Diseases, Emory University, Atlanta, GA, United States; ^5^Johns Hopkins Bloomberg School of Public Health, Baltimore, MD, United States; ^6^Division of Infectious, Diseases, Department of Pediatrics, Emory University, Atlanta, GA, United States; ^7^Department of Anthropology, Emory University, Atlanta, GA, United States; ^8^Center for Tropical and Emerging Global Diseases, University of Georgia, Athens, GA, United States; ^9^Grupo de Investigación Biomedicina, Faculty of Medicine, Fundación Universitaria Autónoma de las Américas, Pereira, Colombia; ^10^Emerging Infectious Diseases and Tropical Medicine Research Group, Sci-Help, Pereira, Colombia; ^11^Master of Clinical Epidemiology and Biostatistics, Universidad Científica del Sur, Lima, Peru; ^12^Department of Microbiology and Immunology, University of North Carolina School of Medicine, Chapel Hill, NC, United States; ^13^Emory Vaccine Center, Yerkes National Primate Research Center, Atlanta, GA, United States; ^14^Georgia Emerging Infections Program, Atlanta, GA, United States

**Keywords:** SARS-CoV-2, ELISA, antibody response, serology, public health

## Abstract

**Background:** Antibodies against SARS-CoV-2 can be detected by various testing platforms, but a detailed understanding of assay performance is critical.

**Methods:** We developed and validated a simple enzyme-linked immunosorbent assay (ELISA) to detect IgG binding to the receptor-binding domain (RBD) of SARS-CoV-2, which was then applied for surveillance. ELISA results were compared to a set of complimentary serologic assays using a large panel of clinical research samples.

**Results:** The RBD ELISA exhibited robust performance in ROC curve analysis (AUC> 0.99; Se = 89%, Sp = 99.3%). Antibodies were detected in 23/353 (6.5%) healthcare workers, 6/9 RT-PCR-confirmed mild COVID-19 cases, and 0/30 non-COVID-19 cases from an ambulatory site. RBD ELISA showed a positive correlation with neutralizing activity (*p* = <0.0001, *R*^2^ = 0.26).

**Conclusions:** We applied a validated SARS-CoV-2-specific IgG ELISA in multiple contexts and performed orthogonal testing on samples. This study demonstrates the utility of a simple serologic assay for detecting prior SARS-CoV-2 infection, particularly as a tool for efficiently testing large numbers of samples as in population surveillance. Our work also highlights that precise understanding of SARS-CoV-2 infection and immunity at the individual level, particularly with wide availability of vaccination, may be improved by orthogonal testing and/or more complex assays such as multiplex bead assays.

## Introduction

Severe acute respiratory syndrome coronavirus 2 (SARS-CoV-2) first emerged in Wuhan, China in December 2019 and rapidly spread to cause an unprecedented pandemic (1). Validated sensitive and specific serologic assays are critical tools for evaluating exposure and immunity to emerging infectious diseases. In the context of SARS-CoV-2, there are multiple uses for tests that detect SARS-CoV-2-reactive antibodies. Epidemiologically, serostatus can be used to track the prevalence and incidence of infection in populations and guide decisions on resource allocation and regulations on public activities (2). Furthermore, measures of SARS-CoV-2-specific antibodies are becoming increasingly important to measure the breadth and durability of vaccine responses, especially with the emergence of novel variant strains.

SARS-CoV-2 is a betacoronavirus that is closely related to other recently emerged coronaviruses (CoVs) SARS-CoV and MERS-CoV and more distantly related to ubiquitous alpha and beta. CoV are spherical, enveloped viruses with large single-stranded positive-sense RNA genomes of ~30 kb (3). The surface of CoV is decorated with homotrimers of the spike (S) glycoprotein that mediate host cell infection via the angiotensin-converting enzyme (ACE-2) receptor on respiratory epithelial cells and represent the primary target of neutralizing antibodies (nAb) (4). CoV infection elicits human antibody (Ab) responses to additional structural and non-structural proteins, with the nucleocapsid (N) being used in serologic assays in addition to S-derived antigens (2).

SARS-CoV-2 infection consistently induces an Ab response in most infected people. The sensitivity for detecting SARS-CoV-2 Ab, including IgM and IgG, peaks at ~3 weeks post symptom onset; however, reports on the durability of SARS-CoV-2 Ab have been variable. Some have reported that Ab responses are transient, with a large proportion of seropositive individuals sero-reverting within a few months. However, detailed longitudinal studies of adaptive immunity to SARS-CoV-2 reveal relatively stable Ab responses through at least 6–8 months after infection (5). Vaccine-elicited Ab responses may be different both quantitatively and qualitatively. Thus far, antibody levels have been reported to be stable up to 6 months post-vaccination (6), although models predict waning of protective immunity after 6 months (7).

The literature and the market were flooded with serologic tests for SARS-CoV-2 Abs in the first several months of the pandemic, including tests with a wide range of readouts, antigens employed, performance and reliability (8). Many tests eventually had their FDA emergency use authorization (EUA) revoked due to quality concerns. This created much uncertainty and confusion surrounding the clinical value of Ab testing (9). However, it has been exceedingly clear that understanding humoral immunity to SARS-CoV-2 infection and developing robust serologic assays is a crucial aspect of the public health response to this pandemic, as well as defining the determinants of protective immunity and developing COVID-19 vaccines (10). Serologic tests intended for clinical use are required to comply with regulatory standards, but variability and poor inter-laboratory agreement can still be a problem (11). Non-clinical assays to detect Ab responses that are used for basic or translational research and epidemiologic purposes often have performance that is less rigorously validated or standardized across laboratories—though efforts to address these issues exist (12). Thus, the goals of this study were to (1) develop and validate a simple, sensitive and specific in-house enzyme-linked immunosorbent assay (ELISA), (2) assess the relative performance of the ELISA in comparison to other robust serologic approaches for measuring SARS-CoV-2 immunity, and (3) determine the advantages and limitations for applying a simple serology assay to address specific research questions. Here we describe our process of developing a useful serological tool amidst the dynamic nature of the early months of the pandemic, in the absence of a gold-standard SARS-CoV-2 antibody assay. We discuss important lessons that remain relevant to the ongoing COVID-19 pandemic and that are also generalizable to the public health response for future emerging viral diseases.

## Materials and Methods

### Human Subjects and Biospecimens

Human specimens (Table 1) were collected from different sources. All data and specimens included in this study were obtained and utilized under protocols approved by the appropriate institutional review boards (IRB), and informed consent was obtained. Specimens collected included serum and plasma by phlebotomy. Saliva was collected using Oracol Plus (Malvern Medical, UK) by brushing the gums for ~1–2 min to obtain gingival crevicular fluid (“saliva” hereafter), which is enriched in serum antibodies and preferred for our assay (13). Specimens were transported to the lab and processed within 24 h and stored at −20° or −80°C. Samples were heat inactivated at 60°C for 30 min prior to use in experiments.

**Table 1:** Characteristics of study population.

**Characteristic**	**Pre-pandemic**		**Cases**	**Ambulatory**	**COPE cohort**
	**“Traveler” sera**	**“Columbian” sera**			
Participants (*N*)	56	84	82	39	353
Sample collection period	May 2018–September 2019	December 2017–April 2019	March 2020–September 2020	March 2020–June 2020	May 2020–June 2020
Median age (range)–year	46 (21–75)	24 (18–41)	48 (23–77)	48 (22–78)	37 (22–71)
**Sex–no. (%)**					
Female	0 (0)	84 (100)	46 (56.1)	23 (57.5)	269 (76.2)
Male	0 (0)	0 (0)	34 (41.5)	17 (42.5)	84 (23.8)
Unknown	56 (100)	0 (0)	2 (2.4)	0 (0)	0 (0)
**Race-no. (%)**					
Asian	0 (0)	0 (0)	6 (7.3)	3 (7.5)	35 (9.9)
Black	0 (0)	0 (0)	22 (26.8)	14 (35.0)	48 (13.6)
Other	41 (73.2)	0 (0)	4 (4.9)	2 (5.0)	18 (5.1)
White	0 (0)	0 (0)	44 (53.7)	20 (50.0)	246 (69.7)
International	7 (12.5)	84 (100)	0 (0)	0 (0)	0 (0)
Unknown	8 (14.3)	0 (0)	6 (7.3)	1 (2.5)	6 (1.7)
Hispanic-no. (%)	0 (0)	0 (0)	2 (2.4)	1 (2.5)	17 (4.8)

#### Pre-pandemic Sera

Two groups of sera from frozen archives were used in this study. “Colombian” sera were collected in a cross-sectional cohort of healthy pregnant women presenting to a Labor and Delivery ward in Risaralda, Colombia between December 2017 and April 2019 (Emory IRB# 103255 and 106096). “Traveler” sera were collected pre- and post-travel from healthy US travelers participating in a surveillance study from May 2018—September 2019 (Emory IRB# 103363) or from healthy subjects who were previously diagnosed with travel-related Zika infection and sampled from June 2018 –May 2019 (Emory IRB# 00022371) (14, 15).

#### COVID-19 Sera

Two groups of patients contributed to this sample set. “Cases” comprise individuals with confirmed SARS-CoV-2 infection by RT-PCR testing, which is the gold standard for diagnosing COVID-19 (acute symptomatic SARS-CoV-2 infection). Subjects in this group were recruited initially from inpatients at Emory University Hospitals beginning in March 2020. In April 2020, confirmed and recovered (convalescent) cases were also recruited at an outpatient research clinic under the same IRB protocol (Emory IRB# 00022371). An “Ambulatory” group comprised patients recruited from an outpatient testing site, all with COVID-19 molecular testing results available (test date range: March 18–June 10, 2020). These participants were symptomatic with mild illness (see Table 1), and these subjects were asymptomatic when convalescent serum was donated (Emory IRB# 110683).

#### COVID Surveillance Sera

“Surveillance” specimens were obtained and allocated for research use as part of a longitudinal surveillance cohort study of healthcare personnel (The COVID-19 Prevention in Emory Healthcare Personnel (COPE) Study, Emory IRB# 00000505). Baseline enrollment for this study was open May 1, 2020, and completed June 12, 2020 (16). Subjects were healthy at time of enrollment and donated serum and saliva.

### Pseudoviruses, Cells, and Key Reagents

Recombinant receptor-binding domain (RBD) protein and the RBD-binding monoclonal antibody CR3022 were used according to previously described protocols (17, 18), as well as SARS-CoV-2 neutralizing monoclonal antibody CC12.1 (19). Plasmids expressing human TMPRSS2 (20), pCMV ΔR8.2, pHR' CMV-Luc (20), and SARS-CoV-2 spike protein (Wuhan-1) were obtained from the Vaccine Research Center at NIAID, NIH (21). Full details for the neutralization assay are in the Supplementary Material.

### ELISA

*SARS-CoV-2 RBD IgG* Thawed serum was used in all ELISA experiments. This assay was developed similarly to previously described protocols (17, 22). Plates were coated with 200 ng/well of recombinant SARS-CoV-2 RBD in phosphate buffered saline (PBS, pH 7.4) at 4°C overnight, then blocked the next morning with 1% BSA (in PBS with 0.05% Tween). A 1:100 dilution of sera in blocking buffer was incubated at 37°C for 1 h and plates washed three times. IgG was detected with goat anti-human IgG conjugated to HRP (1:20,000) at 37°C for 1 h. Plates were developed for 5 min after adding 100 μl o-Phenylenediamine dihydrochloride (OPD) substrate (SIGMA: P8787) with peroxide citrate buffer substrate, and the reaction was stopped with 100 ul 1N HCl. Plates were read immediately at 490 nm. Raw optical density (OD) values were normalized to the absorbance of an internal control [CR3022 mAb used at 2μg/ml (200 ng/well)] and reported as the normalized ratio (NR).

Additional methodology details for the SARS-CoV2 RBD total Ig, IgG3, IgA, IgM, and dried blood spot (DBS) testing are described in the Supplementary Material.

### Saliva Luminex Assay

Saliva swabs were collected and transferred directly to the processing lab. Upon receipt at the lab, swabs were centrifuged at 1,500 g for 10 min to separate the sample from the sponge and then heat-inactivated at 60°C for 30 min. Samples were stored frozen at ≤ -20°C prior to testing. Archived saliva samples that had been self-collected with Oracol swabs as part of different research studies before December 2019 were used as pre-pandemic negative controls. Samples were tested using a modified version of a previously described multiplex SARS-CoV-2 immunoassay based on Luminex technology (13). Further details are in the Supplementary Material.

### Statistical Analysis

#### ROC Curve for RBD IgG ELISA

ROC curve analysis was performed using PRISM Graphpad version 8.4.3 to determine the optimal threshold for the SARS-CoV-2 RBD IgG ELISA (AUC = 0.994).

#### Association Between Ig Isotype Levels and Days Post Symptom Onset

Multivariable linear regression analysis was conducted in PRISM to assess the association between Ig subtype OD_405_ and days post symptom onset when controlling for age and hospitalization status Data transformations were conducted when appropriate to correct for unmet normality and heteroscedasticity assumptions. Interaction and confounding assessment were done to determine the optimal model. Wald p-values and 95% confidence intervals were reported.

## Results

### Validation of RBD IgG ELISA

**Figure 1:**
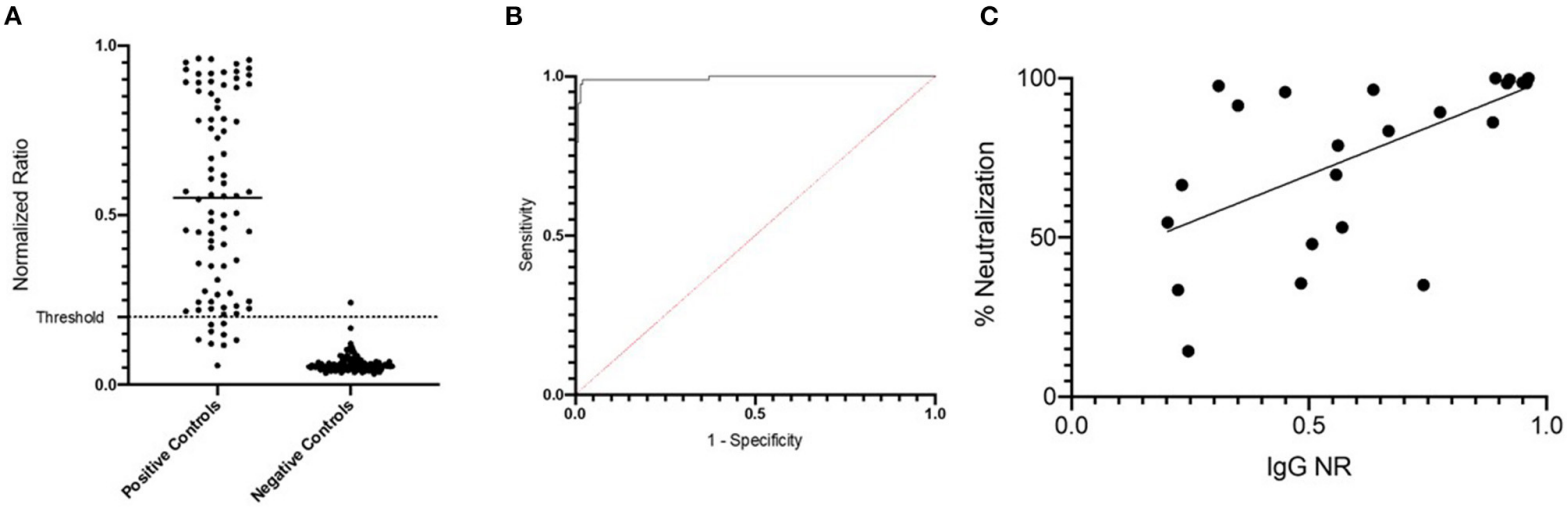
Development and validation of RBD ELISA to detect human SARS-CoV-2-specific IgG. **(A)** Positive and negative control specimens were tested by the RBD ELISA. The normalized ration (NR, see methods) is plotted on the y-axis. The horizontal dashed line at NR = 0.2 indicates the assay cut off as determined by ROC curve analysis shown in **(B)**. **(C)** NR values from the RBD ELISA are plotted (x-axis) against the percent neutralization determined in a screening assay performed at a single dilution (1:30). The screening assay quantifies the amount of pseudovirus infection in the presence of test sera relative to pseudovirus only wells. Values <50% are considered a negative screening test for SARS-CoV-2 neutralizing antibodies.

To establish a simple serologic assay for SARS-CoV-2-specific IgG detection, an ELISA using an RBD antigen was validated by testing a large set of human sera with known infection status (Table 1). Pre-pandemic sera (*n* = 140) constituted negative controls, and positive controls were convalescent sera (10–127 days post symptom onset, DPSO; mean 39.8 DPSO; median 38 DPSO) from RT-PCR-confirmed COVID-19 cases (*n* = 82). The mean normalized ratio (NR) for the Traveler group was 0.05 and for the Colombian group, mean NR was 0.06 (Figure 1A). Thus, the sera from the two cohorts were similarly non-reactive and indicated low background in this assay, with only one of 140 negative controls with a NR >0.2. Sera from convalescent COVID-19 cases showed a mean NR of 0.54, ranging from 0.057 to 0.962 (Figure 1A). The positive control monoclonal antibody (mAb) CR3022, which defined an NR of 1, gave an OD of ~1.5 across multiple plates (data not shown). ROC analysis was conducted to define the cut off that optimized sensitivity and specificity, with priority given to maintaining specificity ≥99% (Figure 1B). A threshold of 0.20 (Sens = 89.0%, Spec = 99.3%) was selected. Percent neutralization was calculated at 1/30 serum dilution and correlated to RBD IgG ELISA normalized ratios (*p* = <0.0001, *R*^2^ = 0.26, Figure 1C).

### Application of RBD IgG ELISA in Ambulatory Patients

To test the hypothesis that serologic testing would increase diagnostic sensitivity for mild COVID-19, convalescent (12–124 DPSO) serum was obtained from patients (*n* = 39) undergoing RT-PCR testing for SARS-CoV-2 infection in an ambulatory clinic. 6/9 (66.7%) RT-PCR-confirmed patients were RBD IgG-positive, and 0/30 (0%) RT-PCR-negative patients tested RBD IgG-positive (Table 2). These results confirm the high performance of molecular diagnostics in symptomatic patients suspected of COVID-19, and we did not identify additional SARS-CoV-2 infections in this small sample set.

**Table 2:** Convalescent serology testing of ambulatory PUI.

	**ELISA+**	**ELISA–**	**Total**
RT-PCR+	6	3	9
RT-PCR–	0	30	30
Total	6	33	39

### Application of RBD IgG ELISA for Surveillance

**Figure 2:**
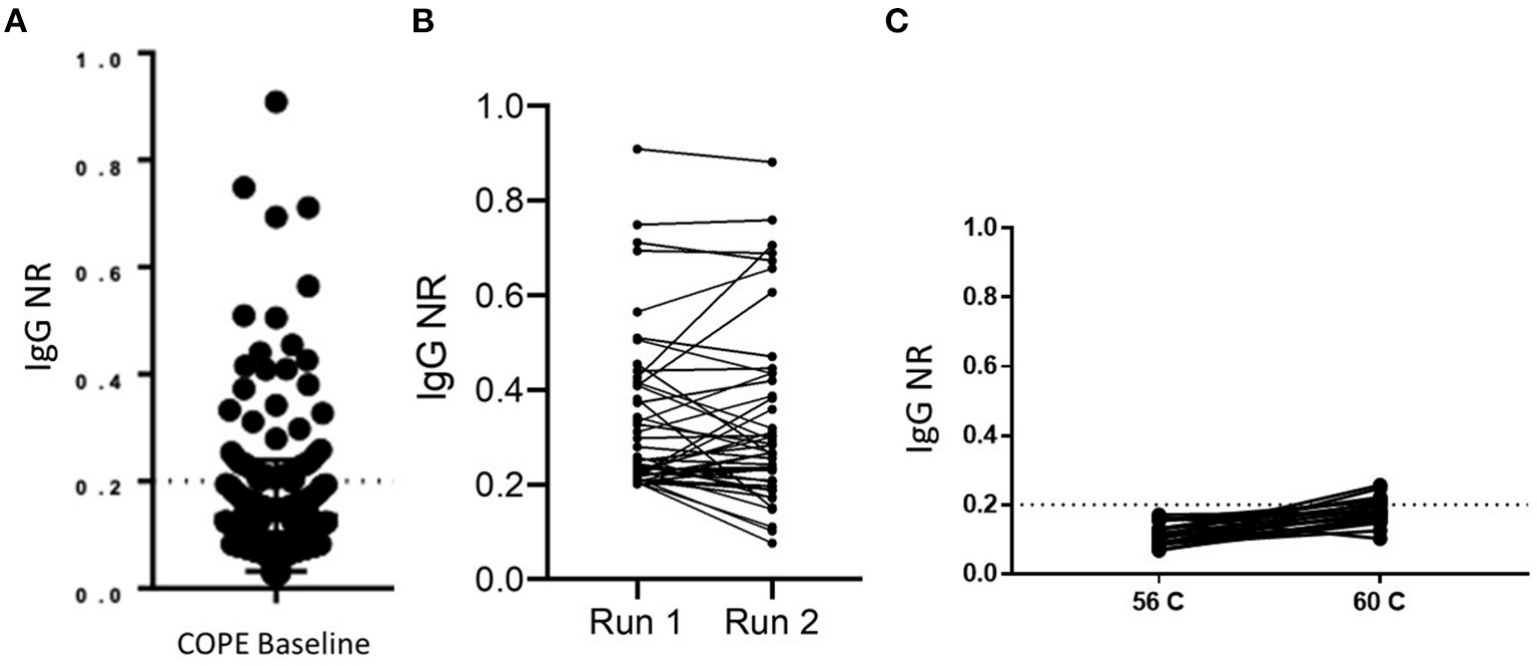
Implementation of RBD IgG ELISA for surveillance. **(A)** The distribution of RBD ELISA results are shown for a cross-sectional sample of 353 healthcare personnel. The dashed line at NR = 0.2 indicates the assay cut off. **(B)** Run-to-run concordance is shown for samples that initially tested positive by RBD ELISA (*n* = 45). **(C)** Paired results in the RBD ELISA are shown for negative control samples which had one aliquot heat inactivated at the indicated temperature (*n* = 21).

SARS-CoV-2 seroprevalence was determined by RBD ELISA in a cohort of healthcare personnel (HCP) in Atlanta, GA, following baseline enrollment that occurred from May 5 to June 12, 2020. This result of 23/353 RBD ELISA positive [6.5% seroprevalence)] was previously reported (16). The distribution of NR values is shown in Figure 2A. Because many samples had results very close to the cut off, we required samples to test consistently positive on at least two independent runs of the assay to confirm positive status. By these criteria, 23 sera were confirmed as positive of the 45 identified as close to the cut off on a first run. There was variability (mean %CV 23.8%) in ELISA results between multiple test runs (Figure 2B); however, only one sample with a NR of ≥ 0.25 was rejected upon repeat testing. Of note, <2% (7/353) of the HCP cohort reported a positive RT-PCR swab prior to baseline sample collection. Five of the seven participants with a positive RT-PCR tested positive by RBD ELISA. The two that tested negative had a NR of 0.144 and 0.027. Given the number of samples giving an NR close to the assay cut off, we questioned whether sample-intrinsic background signal may be an issue, particularly because lab safety policy required heat inactivation of samples at 60°C rather than the standard 56°C, and concern for this practice in affecting both sensitivity and specificity of serologic assays has been raised. We analyzed 21 samples by running an aliquot that was and was not heat inactivated at 60°C side-by-side on the RBD ELISA. The variation in results was similar to what was observed for testing the same sample on multiple plates, with only a minimal increase in mean NR of 0.115–0.183 (Figure 2C).

### Comparative Performance of RBD ELISA to Alternative Serodiagnostic Assays

In addition to rigorous validation with control specimens, we sought to compare results from the RBD ELISA assay with additional well-established assays. We tested a subset of selected HCP surveillance samples and controls, with the results of the orthogonal testing shown in Table 3 (additional details in Supplementary Material: orthogonal testing). We titrated IgG levels with the same RBD ELISA and examined the correlation among endpoint titer and NR at the 1:100 dilutions. We also performed ELISA testing for total Ig (23) and found good agreement in results. Discrepant results between these two assays typically involved a lower signal in the total Ig assay that was more consistent with multiplex saliva and serum testing. We have previously shown that detection of SARS-CoV-2-specific IgG in saliva on a Luminex platform is robust (~100% accurate) and closely matches results obtained in matched serum run in the same Luminex assay (13). Salivary antibody testing detected SARS-CoV-2 IgG in 15 of 39 of RBD ELISA positive samples giving a positive percent agreement (PPA) of 37.5% (Supplementary Table 2). The PPA between these two assays increased to ~60% when only considering RBD IgG ELISA+ samples with an NR≥0.25. Negative agreement was strong among these two assays (and for all assays). Of 40 samples testing negative in the RBD IgG ELISA (NR <0.2), 37 resulted negative in the saliva assay (NPA=92.5%). NPA agreement increased to 96.3% when considering the 27 samples with RBD IgG ELISA NR <0.18. We also tested sera from this sample set in a distinct Luminex assay that employs in-house SARS-CoV-2 antigens produced in an *E. coli* expression system. Again, concordance of results was observed for assay positivity as well as relative order of signal magnitude among positive samples (Table 3). Finally, a single dilution (1:30) neutralization screening assay (NSA) was performed to assess whether our binding ELISA test predicted antibody function (Table 3). RBD ELISA testing agreed well with NSA in positive control and COPE samples, particularly at higher NR values. Agreement was more variable in low to intermediate positive samples.

**Table 3:**
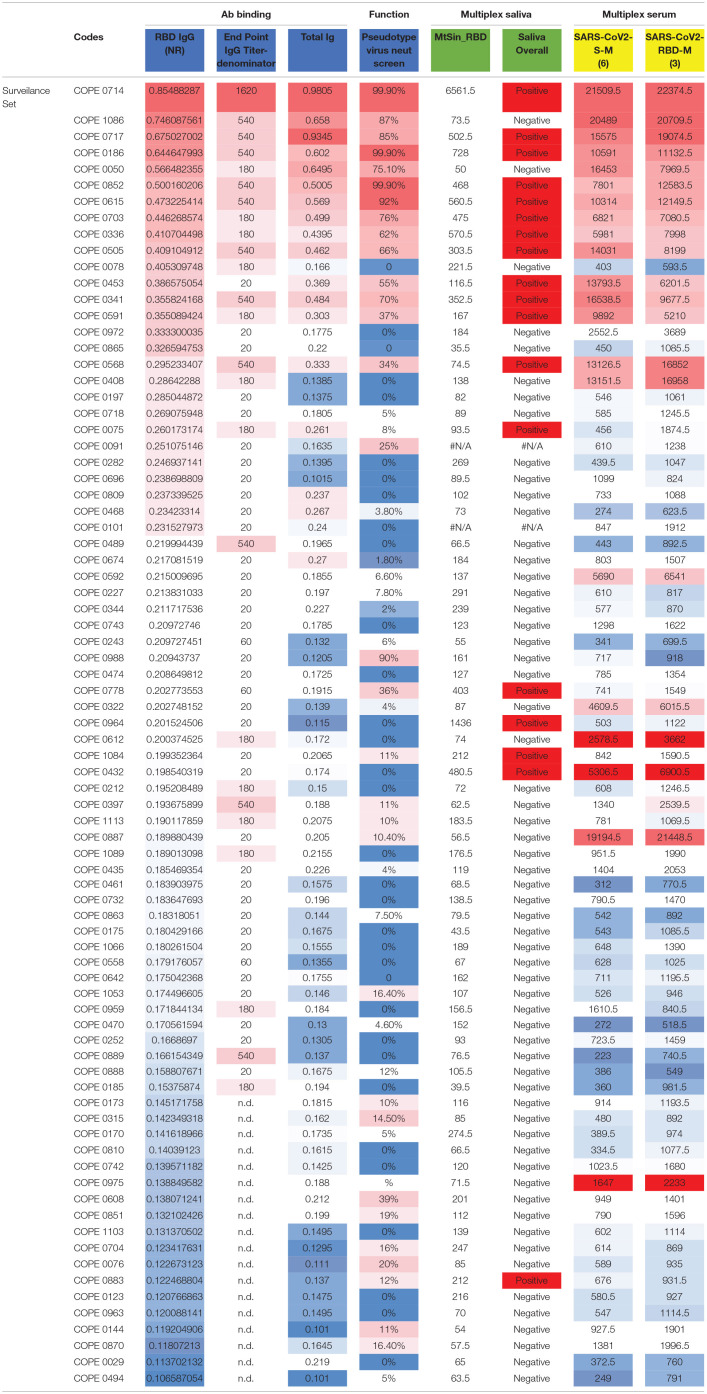
Orthogonal testing.

### Association of Antibody Subtypes and Isotypes With Days Post Symptom Onset

**Figure 3:**
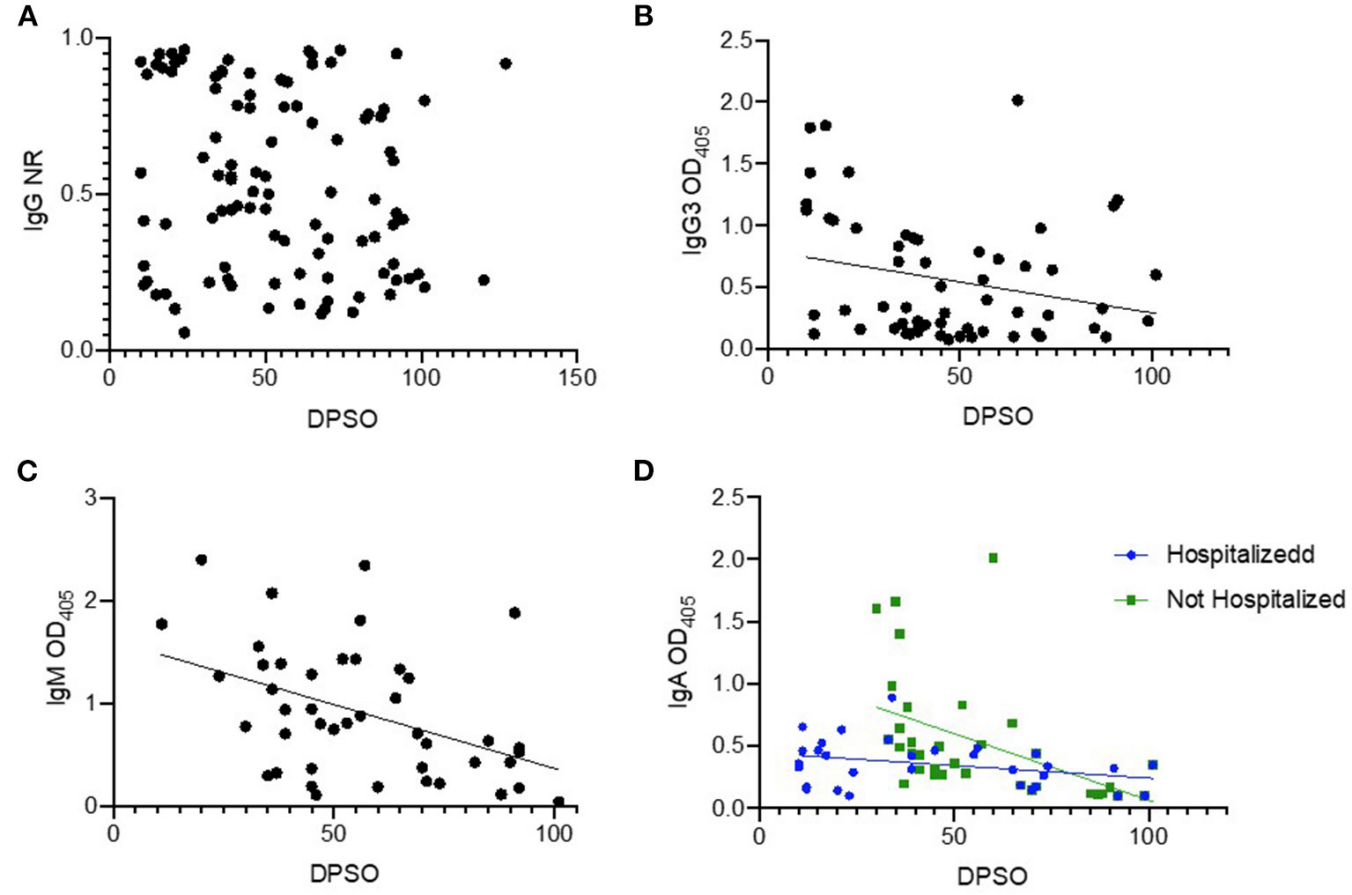
Temporal relationships between time of infection and antibody isotypes. Cross-sectional samples were tested by ELISA for the indicated antibody isotype or subtype, and magnitude of signal was plotted (y-axis) against the days post symptom onset (DPSO, x-axis). Samples (*n* = 100) from RT-PCR-confirmed cases (*n* = 82) were used in these analyses. A few subjects had a sample from multiple time points available, and these are included as un-linked independent data points for this analysis. All ELISA assays are antigen coating indirect ELISAs using RBD as the antigen. The secondary antibody for IgG detection is conjugated to HRP and the normalized ration (NR) is reported as described in methods **(A)**. The secondary antibodies for IgG3, IgM and IgA use alkaline phosphatase, results reported are mean optical density (OD) of technical replicates **(B–D)**.

We hypothesized that certain immunoglobulin (Ig) subtypes may allow for refining the timing of SARS-CoV-2 infection in cross-sectional surveillance efforts. In addition to IgG, we analyzed IgG3, IgA, and IgM with respect to DPSO while controlling for potential confounders such as severity of illness and patient age (Figure 3). We were not able to assess the presence of an association between DPSO and IgG because normality assumptions could not be met using common data transformations (Figure 3A). No relationship was found between IgG3 OD_405_ and DPSO after controlling for patient age and hospitalization status (Figure 3B). DPSO was associated with IgM OD (*p* =0.0027) with no evidence of confounding or effect measure modification by other variables of interest (Figure 3C). There was a significant negative correlation between DPSO and IgA OD (*p* = < 0.0001). Hospitalization status was a significant effect measure modifier in this relationship (*p* = 0.0004), with patients with milder disease exhibiting stronger IgA responses in early convalescence that declined over time. Log transformation was used to in the model assessing IgA and DPSO and a square root transformation was used to in the model assessing the relationship with IgG3 and DPSO to correct for unmet normality homoscedasticity assumptions.

### Performance of RBD IgG Assay in Different Diagnostic Specimen Types

**Figure 4:**
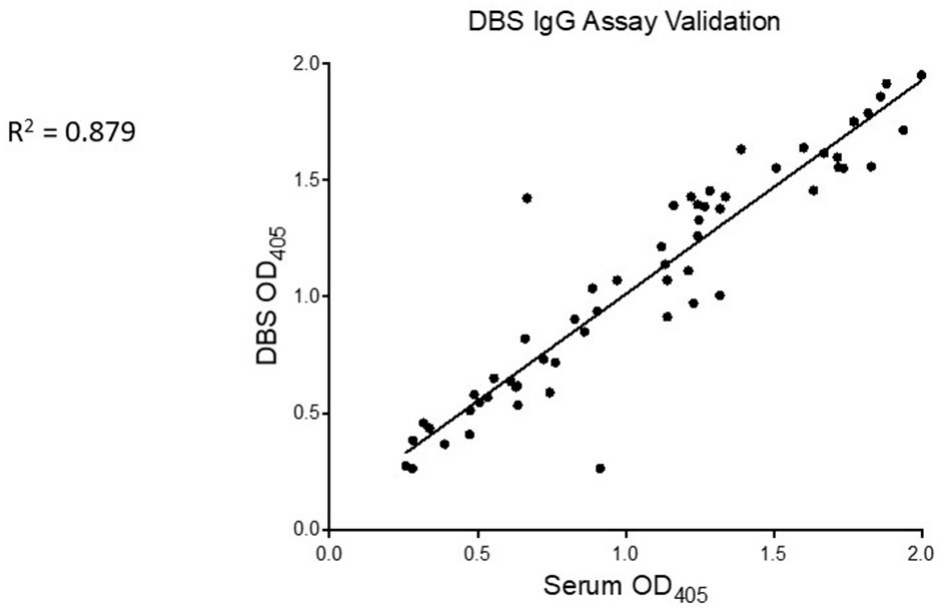
SARS-CoV-2-specific IgG is similarly detected from serum and DBS. Serum (x-axis) and eluate from control DBS (y-axis) were run side-by-side on RBD ELISA plates. Mean OD of technical replicates was plotted on XY scatter graph in Prism and a linear regression analysis was performed. Pearson correlation coefficient and p value are displayed on graph.

In a subset of the positive controls (*n* = 59), we tested for SARS-CoV-2-binding IgG by the RBD ELISA in serum and dried blood spot (DBS) eluate, running matched specimens from the same individual side-by-side on the same ELISA plate. There was a strong linear correlation in NR values for these two specimen types (*p* < 0.001, *R*^2^ = 0.879, Figure 4).

## Discussion

In this study, we rapidly developed a useful serologic assay during the early months of the ongoing COVID-19 pandemic, which was then applied in unique populations for testing and surveillance. We validated a SARS-CoV-2 RBD IgG ELISA by the traditional approach of control sample testing and ROC curve analysis, which indicated robust performance parameters and a sensitivity of 89% and specificity of 99.3% for samples collected at least 10 days after symptom onset. We also pursued a more extensive examination of performance via orthogonal testing by multiple Ab detection platforms, which generally showed concordant patterns of results.

We noted some variability existed among the different tests, most notably for samples with borderline NR values in the RBD IgG ELISA. Among samples with discrepant RBD ELISA and NSA results, comparing to a third or fourth test platform was typically clarifying. However, there are exceptions, for example, COPE0078 is likely a false positive on the ELISA RBD assay as it has a completely negative NSA and low signal on multiple SARS-CoV-2-specific antigens on the serum Luminex assay. Additional cross-assay comparisons are interesting. For example, COPE0778 is positive right at the cutoff of the RBD IgG ELISA, has low signal in the total Ig and NSA but also tests positive by saliva assay. Interestingly, the serum multiplex reveals low signal for S, RBD, and N, but a strong signal for Orf8, suggesting that a small portion of SARS-CoV-2 infected individuals may exhibit immune-dominance patterns that are not focused on S and N antigens, which could lead to misclassification of SARS-CoV-2 infection status in a small proportion of people by many serologic tests (24). Similarly, COPE0887 exhibits a negative RBD IgG ELISA, a negative NSA and negative testing by saliva assay; however, the serum Luminex assay detected strong reactivity, particularly to N, orf3 and orf8 antigens, with much more modest signal to RBD and S. Others have noted SARS-CoV-2-specific responses to orf3 and orf8 (24, 25), which may enable detection of Ab against SARS-CoV-2 in individuals that do not mount strong responses to the immunodominant S or N antigens, but simple assays based on orf3 or orf8 antigens may not be sufficiently sensitive. Overall, these data indicate convergent results of Ab testing for a high proportion of samples from individuals with prior SARS-CoV-2 infection and lend validity to the simple RBD IgG ELISA as a pragmatic testing approach to determine SAR-CoV-2 immunity.

Serologic testing will remain a critical element of the public health response to the COVID-19 pandemic for the foreseeable future. Robust assays, particularly those linked to functional activity or correlated with immunologic protection as is true for RBD ELISAs (19), are essential for assessing population level prevalence and incidence of SARS-CoV-2 infection, as well as for determining infection endpoints in intervention trials. With the impending FDA full-licensure of current SARS-CoV-2 vaccines and development of novel vaccines, serology, and immune correlates will also be essential to translate laboratory values to clinical relevance. The role of serology in clinical care has been limited to date but may become increasingly important for specific scenarios, such as in patients experiencing post-acute sequelae of SARS-CoV-2 infection (PASC or “long COVID”) who have only mild or completely absent symptoms at the time of initial infection (26). We did not detect cases of false negative SARS-COV-2 molecular testing in our small ambulatory sample set (*n* = 30), which is consistent with high sensitivity of these assays. However, serology may marginally improve sensitivity for case identification, and this could be most important when certain antigen tests with moderate sensitivity are being used rather than RT-PCR for diagnosing symptomatic individuals (27). Moreover, if SARS-CoV-2 transmission decreases and becomes one of many potential etiologies for prolonged critical respiratory illness, complementing molecular testing with high performing serology tests may clarify diagnoses and impact management. Incorporating multiple Ig subtypes as we have studied to aid in timing the infection could enhance the utility of serologic testing.

Although there are currently numerous SARS-CoV-2 diagnostics available, it is important to acknowledge the heterogeneity of these assays and determine which tests are best implemented in different contexts such as individual infection categorization vs. population-level surveillance. Unfortunately, the diagnostic landscape has been further complicated by distribution of low performing assays, which culminated in the FDA revoking several EUA that had been previously granted (28). Although current diagnostics have described sensitivity and specificity values for known positive and negative cases, there is little data that describes application of ELISA-based SARS-CoV-2 diagnostics in subjects with unknown exposures or in those with intermediate results. Our study extends existing data on use of SARS-CoV-2 Ab tests by examining the RBD IgG ELISA characteristics in a surveillance setting for healthcare workers who were asymptomatic when sampled and in a group of mildly symptomatic patients being tested for COVID-19.

As seen with other pandemics, public health systems in lower and middle-income countries (LMICs) may lack the means to adequately respond to the emergence of SARS-CoV-2 (29). Insufficient capacity for surveillance is one of many concerns for regions in Africa and Latin America (30, 31). Even in wealthier nations, vulnerable and underserved populations have been disproportionately impacted by COVID-19, which is partly attributable to lack of access to diagnostic testing. For example, major outbreaks have occurred in the US corrections system, leading to delayed diagnoses. Several aspects of our work address the challenges of SARS-CoV-2 surveillance and testing, primarily the need for needle-free testing. We increasingly believe that saliva-based assays are an attractive strategy to maximize sampling and access of SARS-CoV-2 Ab testing for public health purposes (13, 32). Furthermore, we show that DBS offers another sampling strategy that exhibits high fidelity to phlebotomy-based specimens and can be utilized in resource poor settings with limited refrigeration or advanced laboratory equipment. Deployment of sampling approaches such as saliva finger stick DBS are both further supported in that these are highly amenable to self-collection and mail-in for analysis by a central lab. Finally, prokaryotic antigen expression systems such as was used here for certain antigens in the serum Luminex assay represent a relatively simple technique that could be established in labs in resource-limited settings if not already available. Interestingly, Luminex has an installation base of >10,000 machines globally, which includes many health centers or government labs in LMICs. Thus, many elements of this study are based on methodologies that are readily scalable to support broad implementation of serologic testing worldwide.

A limitation of our study was the modest sample size of the ambulatory population. These results should be interpreted with caution when generalizing to other patients with mild symptoms. It should also be noted that nearly all the cases used for assay validation were symptomatic. It is not clear how sensitive the assay is in detecting asymptomatic infection. For the longitudinal sample assessments, not all participants enrolled had multiple or consistent time points measured. For the healthcare worker cohort, only a single time point was measured. Prior studies have demonstrated waning antibody levels and even sero-reversion (33). Further analysis is warranted to capture additional time points for kinetic studies. Additionally, we focused mainly on anti-RBD IgG in both sera and saliva. Higher sensitivity and specificity may be gained with a combination of antibody assays that target different components of the SARS-CoV-2 virus. Furthermore, ratios of RBD antibodies to nucleocapsid antibodies may provide further characterization or prediction of illness severity (34). For the DBS analysis, only a limited number of samples were analyzed, with good linear correlation. To describe the sensitivity and specificity of this assay and the relationship with serological findings, further studies with a larger sample size and pre-pandemic controls should be analyzed.

In summary, this study demonstrates the applied utility of simple in-house ELISA testing for SARS-CoV-2-reactive IgG, which could be deployed to labs in most parts of the world. The collaborative process by which we developed, validated, and implemented the assay during the pandemic is a model by which future serologic assays can be designed in the setting of emerging pathogens. Data shown here are also support the idea that access to serologic testing could be expanded by implementing alternative sampling strategies such as saliva or DBS that do not require phlebotomy. Our study also highlights the value of orthogonal testing in defining the true status of a minority of samples with discrepant test results. However, the benefit of single assay approaches—provided the performance is stringently established—will likely outweigh the more complete and accurate assessment of individual sero-status of multi-platform orthogonal testing by offering sufficient accuracy and better throughput at lower cost for meeting demands of testing volume, including in resource-limited settings.

## Data Availability Statement

The original contributions presented in the study are included in the article/Supplementary Material, further inquiries can be directed to the corresponding author/s.

## Ethics Statement

The studies involving human participants were reviewed and approved by Emory Institutional Review Board (IRB). The patients/participants provided their written informed consent to participate in this study.

## Author Contributions

AS was involved in the study design, study procedures, analysis, and drafted the manuscript. TS, KT, SH, and TL provided statistical support and analysis and critically reviewed the manuscript. YZ, DE, TWS, and JK performed laboratory analyses and critically reviewed the manuscript. JH-A and SF led the epidemiological cohort and critically reviewed the manuscript. NR oversaw study procedures and aided in the clinical study design and critically reviewed the manuscript. SH, RT, NP, CH, JW, LP, and ES provided laboratory expertise and analysis and critically reviewed the manuscript. MC conceptualized the study, directed the laboratory analysis, study procedures, study design, and crucially reviewed the manuscript. All authors contributed to the article and approved the submitted version.

## Funding

AS was supported by the NIH Vaccinology training grant (T32AI074492). Support for specimen collection and processing of COPE samples was provided by the Georgia Emerging Infections Program, which was funded through the Centers for Disease Control and Prevention Emerging Infections Program [U50CK000485]. JH-A was supported by the Antibacterial Resistance Leadership Group fellowship (National Institute of Allergy and Infectious Diseases UM1AI104681). RT and HS were supported by grant R01AI125738 from the National Institutes of Health and a University of Georgia Athletic Association endowment to RT. Development of multiplex saliva testing was supported by an NIH grant to CH and MC (R21AI139784).

## Author Disclaimer

The content is solely the responsibility of the authors and does not necessarily represent the official views of the National Institutes of Health or other funding agencies.

## Conflict of Interest

The authors declare that the research was conducted in the absence of any commercial or financial relationships that could be construed as a potential conflict of interest.

## Publisher's Note

All claims expressed in this article are solely those of the authors and do not necessarily represent those of their affiliated organizations, or those of the publisher, the editors and the reviewers. Any product that may be evaluated in this article, or claim that may be made by its manufacturer, is not guaranteed or endorsed by the publisher.
