# Supplementary material for: Application of SARS-CoV-2 Serology to Address Public Health Priorities
Source: Front Public Health. 2021 Nov 23;9:744535. doi: 10.3389/fpubh.2021.744535 (PMC8650110; doi:10.3389/fpubh.2021.744535)
Supplement: Supplementary file 1 [file Data_Sheet_1.PDF]

Supplemental Material

Study Flowchart

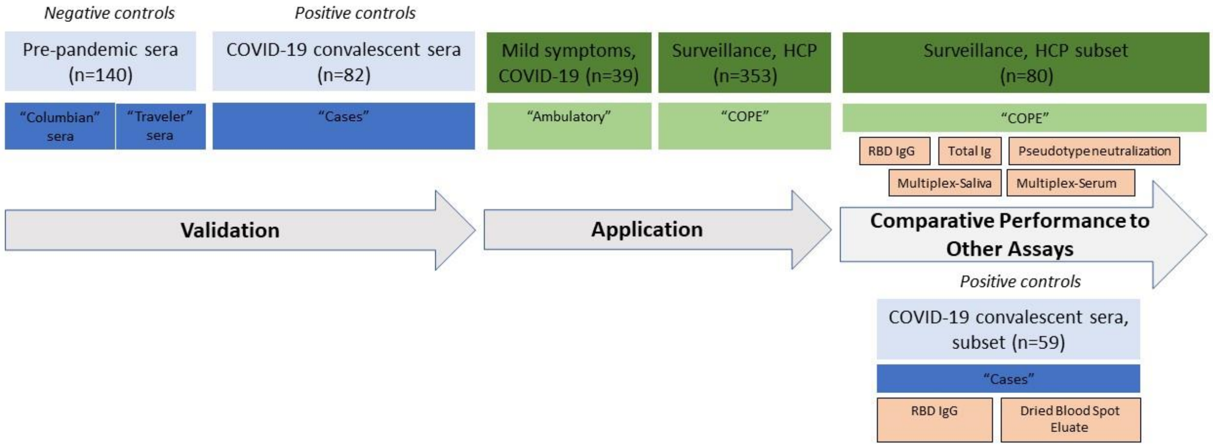

Methods

SARS-CoV-2 ELISA

SARS-CoV-2 RBD Total Ig Briefly, 50 µl of RBD antigen at 4 µg/ml in Tris Buffered Saline (TBS) pH 7.4 was coated in the 96-well high-binding microtiter plate (Greiner bio one cat # 655061) for 1 hr at 37°C using previously described methods [5]. Then the plate was washed three times with 200 µl of wash buffer (TBS containing 0.2% Tween 20) and blocked with 100 µl of blocking solution (3% milk in TBS containing 0.05% Tween 20) for 1 hr at 37°C. The blocking solution was removed, and 50 µl of serum sample at 1:20 or indicated dilutions in blocking buffer was added for 1 hr at 37°C. The plate was washed in the wash buffer and 50 µl of alkaline phosphatase-conjugated secondary goat anti-human secondary Ab at 1:2500 dilution was added for 1 hr at 37°C. For measuring total Ig, a mixture of anti-IgG (Sigma Cat # A9544), anti-IgA (Ab cam Cat # AB97212), and anti-IgM (Sigma Cat # A3437] were added together. The plate was washed, and 50 µl P-Nitrophenyl phosphate substrate (SIGMA FAST, Cat No N2770) was added to the plate and absorbance was measured at 405nm using a plate reader (Biotek Epoh, Model # 3296573).
SARS-CoV-2 RBD IgG3 This assay was performed as for the IgG assay with the following modifications: Sera were diluted 1:20. The secondary antibody (goat anti-human IgG3-HRP, MilliporeSigma) was used at 1:2000). Plates were read at 490 nm. A serum was identified among the COVID-19 cases with particularly strong reactivity and included on each plate as a calibration control.
SARS-CoV-2 RBD IgA This assay was performed as with IgG with the following modifications: Sera were diluted 1:50. The secondary antibody (goat anti-human IgA-AP) was used at 1:2500. The substrate for this enzyme is *p*-nitrophenyl phosphate substrate (MilliporeSigma). Plates were incubated with substrate for up to 20 min at room temperature then read at 5 min intervals at 405 nm. A strongly reactive serum was included on each plate as a calibration control.

SARS-CoV-2 RBD IgM The IgM ELISA was adapted from previously published methods [19]. Nunc Maxisorb flat bottom 96-well plates (ThermoFisher) were coated with 100 µL of recombinant SARS-CoV-

2 RBD at 3 µg/mL in PBS and incubated overnight at 4°C. The next morning, plates were washed four times with 1X PBS containing 0.05% (v/v) Tween20 (PBS-T) in a 96 well plate washer (Biotek ELx405) and blocked with 200 µL of 1% BSA (w/v) in PBS-T (ELISA buffer) for 1.5 hours at room temperature (RT). The blocking solution was removed and 100 µL of serum dilutions in ELISA buffer were incubated for 1.5 hours at RT. Serum samples were run at a 1:100 dilution. The plates were washed four times with PBS-T and 100 µL of HRP-conjugated rabbit anti-human IgM (Jackson ImmunoResearch) at 1:500 dilution in ELISA buffer were added and incubated for 1.5 hours at RT. The plates were washed four times with PBS-T as above and once with 100 µL of 1X PBS by hand. HRP substrate (100 µL of O-Phenylenediamine dihydrochloride in 0.05 M phosphate-citrate buffer with H_2O_2 (Sigma)) was added and allowed to develop for 5 minutes at RT. The reaction was stopped with the addition of 100 µL 1N HCl and absorbance values at 490 nm measured by a plate reader (Biotek Synergy NeoS2). A positive control serum was used for each experiment, as well as a negative control human serum pooled from six individuals, where the individual negative control sera did not exhibit reactivity against SARS-CoV-2 RBD or spike.

Dried blood spot testing

Dried blood spots (DBS) were created in the lab by transferring 45mL of whole blood (collected in a tube containing EDTA) to a Whatman protein saver card. Cards were dried for at least 2 hours or overnight in a biosafety cabinet at room temperature and stored in Ziplock sealed plastic bags at 4°C with abundant desiccant packets. For testing, proteins were eluted from DBS as previously described [26–28]. One 6-mm hole punch of each DBS was placed in a 1.5 mL Eppendorf tube with 300 [illegible]L of PBS and incubated with automated mixing for 2 hours at 37°C. This yielded an eluate that is equivalent to a 1:40 dilution. Eppendorf tubes were centrifuged then eluate transferred to a new tube. Eluate was heat inactivated for 30 minutes in a 60°C water bath. The samples were centrifuged again to pellet proteinaceous debris and the supernatant was transferred to a new tube and stored at 4°C for up to 1 week or at -20°C until use.

Neutralization Assay

Serum neutralizing activity was measured against SARS-CoV-2 pseudoviruses constructed from HIV-1 lentiviruses carrying luciferase reporter genes and pseudotyped with full-length SARS-CoV-2 spike protein (SARS-CoV-2 pseudoviruses). The following neutralization assay was adapted from previously published methods [23]. Pseudoviruses were produced by seeding 16 million 293T cells into 16 ml DMEM with 10% heat-inactivated fetal bovine serum (FBS), 1x GlutaMAX (ThermoFisher), and 1% penicillin/streptomycin (DMEM-10) in a T-150 flask approximately 24 hours prior to transfection. On the day of transfection, an HIV-1 lentiviral packaging plasmid, pCMV R8.2 (17.5 ug); luciferase reporter plasmid, pHR' CMV-Luc (17.5 ug); plasmid expressing SARS-CoV-2 full length S protein (Wuhan-1) (1 ug); and plasmid expressing human TMPRSS2 (0.3 ug) were co-transfected into 293T cells (ATCC CRL-3216) using FuGENE 6 (90 ul) as a transfection reagent (Promega) in Opti-MEM I reduced-serum media (ThermoFisher, 910 ul). Forty-eight hours after transfection, cell supernatant with SARS-CoV-2 pseudoviruses was removed, clarified from cell debris by brief centrifugation (250xg), filtered (0.45 um), and stored in aliquots at -80°C until use.

Prior to SARS-CoV-2 pseudovirus titration, ACE2-HeLa cells [21] were seeded in Nunc Edge 2.0 plates (ThermoFisher) at 5,000 cells per well in DMEM-10 (100 ul). Approximately 24 hours later, DMEM-10 media was removed by gently tapping out media into waste and drying on towels and 50 ul of diluted

SARS-CoV-2 pseudoviruses (2-fold dilution series starting at 1/2 dilution of SARS-CoV-2 pseudoviruses) in MEM medium with 5% FBS), 1x GlutaMAX, and 1% penicillin/streptomycin (MEM-5) were added in octuplicate onto ACE2-HeLa cells and incubated for two hours at 37°C. After two hours at 37°C, 150 ul MEM-5 media was added and plates incubated an additional 72 hours at 37°C. After 72 hours, media was again tapped out of wells, rinsed gently with 150 uL PBS, tapped out again, and 25 ul 1x Luciferase Cell Culture Lysis Reagent (Promega) added to wells with shaking for 15 minutes at room temperature. After shaking, 20 ul of lysate was added to 96-well black and white isoplates (Perkin-Elmer) and 50 ul luciferase substrate (Luciferase Assay System, Promega) added using an injector on a Biotek Synergy Neo2. Luminescence in plates was measured using a 2 second delay and a 1 second integration time with a gain of 170 (average of highest signal wells – virus only). The mean background signal (average of signal in cells only wells) was subtracted from the signal of wells with pseudoviruses prior to determining mean pseudovirus relative luciferase units (RLUs). The final dilution of SARS-CoV-2 pseudoviruses yielding 100,000-200,000 RLUs was selected for future experiments.

For the neutralization assay, the same protocol was followed as for the titer above, except that pseudoviruses at 2X final dilution were mixed with equal parts serum dilution prior to incubating on HeLa cells for 2 hours at 37°C. Serum dilutions were 1/15 (to achieve 1/30 final dilution). Mean background signal was subtracted from signal of wells containing pseudovirus or pseudovirus plus serum. Percent neutralization was determined using the following formula: 100 x (Mean background-subtracted pseudovirus signal – Mean background-subtracted pseudovirus + serum signal)/(Mean background-subtracted pseudovirus signal). Serum samples that did not achieve 50% neutralization were recorded as having a reciprocal titer of <30. The SARS-CoV-2 neutralizing monoclonal antibody CC12.1 (1 ug/ml) was used as a positive control for each experiment, as well as a negative control human serum pooled from six individuals, where the individual negative control sera did not exhibit reactivity against SARS-CoV-2 RBD or spike.

<u>Saliva Luminex</u>

The modified multiplex assay version was composed of 23 unique magnetic beads sets (MagPlex microspheres), instead of 12 sets. Each set was coupled covalently with antigen, antibody or BSA (controls) as described previously (5 µg protein per 1 million beads) [15]. The multiplex test included SARS-CoV-2 nucleocapsid (N), receptor binding domain (RBD), spike (S) antigens, SARS, MERS, RSV, human coronavirus E229, NL63, HKU1, and OC43 antigens in addition to control antibodies and proteins (BSA, anti-human IgG, IgM, IgA antibody; see **Table S1**). Saliva was tested for SARS-CoV-2 IgG and IgG binding to additional virus antigens in the multiplex assay as described previously7. Briefly, saliva was thawed and centrifuged for 10 minutes at 20,000 g. Then 10 µL saliva supernatant was added to a 96-well microtiter plate containing 40 µL PBST with 1% BSA (assay buffer) and 1,000 coupled beads per bead set in each well. Each plate contained 1-2 blank wells with assay buffer instead of sample that were used for background fluorescence subtraction. Positive control saliva was created by spiking SARS-CoV-2 IgG positive saliva with high IgG signal to SARS-CoV-2 antigens into pre-pandemic negative control saliva. The same pre-pandemic saliva was used as negative control. Phycoerythrin-labeled anti-human IgG diluted 1:100 in assay buffer was used to detect the IgG signal in saliva (**Table S1**). The plate was read on a Luminex MAGPIX instrument.

Total salivary IgG ELISA for saliva sample QC The total IgG concentration in saliva was determined using Salimetrics Salivary Human Total IgG ELISA Kits according to the manufacturer's instructions with two

modifications. The incubation times with diluted saliva sample and with detect antibody were reduced to 1 hour instead of 2 hours. This modification has been approved by the manufacturer. Standards and high and low IgG controls with defined total IgG concentration ranges were included on each plate.
For proteins produced in *E. coli*, codon optimized expression constructs in pET30a vector between BamH I and Hind III sites with an in-frame N-terminal 6X-histidine metal affinity tag were ordered from GenScript, Piscataway, NJ). Protein production, purification and attachment to Luminex beads and conduct of the Luminex assay was essentially as described previously with serum/plasmid diluted 1/100 [25]. Mammalian cell-produced proteins Spike-m and RBD-m were donated by Dr. Mark Tompkins, Univ of Georgia. Data are expressed as raw mean fluorescence units from a minimum of 200 beads per determination.
Salivary multiplex assay statistical analysis The blank-subtracted (net) median fluorescence intensity (MFI) was used for statistical analyses. Cutoffs to discriminate SARS-CoV-2 IgG positive from IgG negative samples for each SARS-CoV-2 antigen were defined as the average net MFI plus three standard deviations of a subset of negative control samples. The resulting sensitivity and specificity of SARS-CoV-2 antigens was calculated using pre-COVID-19 saliva samples (negatives) and saliva samples that were collected by participants in the COVID-19 ambulatory study (positives). In addition to relying on single antigens to determine presence of IgG to SARS-CoV-2, we explored several algorithms and combinations of antigens including binary (positive/negative) and continuous classification variables with their own thresholds. Algorithms investigated included: IgG positive samples are defined by an IgG signal above the cutoff (signal to cutoff ratio [S/CO]) to at least one nucleocapsid and one RBD or S antigen (Algorithm No. 1); two or more RBD or spike (S) antigens (No. 2); either algorithm (1) or (2) (No. 3). Additional algorithms explored using signal to cutoff ratios to multiple antigens and a threshold based on pre-pandemic negatives applied to the sum of these select antigens in the multiplex assay: only N antigens (n=2; Alg. No. 4), only RBD antigens (n=3; Alg. No. 5), only S antigens (n=2; Alg. No. 6) and to combinations of N, RBD and S antigens (n=7 or n=6; Alg. No. 7 and 8). Lastly, we explored whether normalizing the SARS-CoV-2 IgG signal by the total IgG (tIgG) concentration in ug/mL in the saliva sample measured by ELISA (Alg. No. 9 and 10) would improve sensitivity and specificity. The test performance was then optimized by applying minimum sample quality control (QC) measure based on the total salivary IgG concentration within each saliva sample. A minimum total IgG concentration requirement was 15 µg/mL. The minimum total IgG requirement was only applied when samples tested negative using the applied algorithm, i.e. samples testing positive for SARS-CoV-2 IgG were not subject to the minimum total IgG concentration requirement, whereas samples that tested negative did not pass QC if their minimum total IgG concentration was under the minimum concentration defined. The number of samples lost due to this requirement was also calculated.

Supplemental Tables

Table S1. Antigens and controls used to create the multiplex assay and secondary antibody.

No.	Vendor / Producing Lab	Antigen/Antibody	Cat. No.
1	Native Antigen	Nucleoprotein	REC31851
2	GenScript	Nucleoprotein	Z03480
3	Sino Biological	Spike S1 RBD (1)	40592-V08H
4	Sino Biological	Spike S1 RBD (2)	40592-V08H
5	Mt. Sinai	RBD	n/a
6	GenScript	RBD (h)	Z03483
7	Mt. Sinai	Whole Spike	n/a
8	Sino Biological	S1+S2 ECD	40589-V08B1
9	Native Antigen	S1	REC31806
10	Native Antigen	SARS 2002 N	REC31744
11	Sino Biological	SARS 2002 RBD	40150-V08B2
12	Native Antigen	MERS S1	REC31760
13	Sino Biological	hCoV NL63 S1+S2 ECD	40604-V08B
14	Sino Biological	hCoV 229E S1+S2 ECD	40605-V08B
15	Sino Biological	hCoV OC43 HE	40603-V08H
16	Sino Biological	hCoV HKU1 S1	40021-V08H
17	Sino Biological	RSV RSS2	40037-V08B
18	Sino Biological	RSV A2	11049-V08B
19	Jackson ImmunoResearch	anti-IgG	109-005-098
20	Jackson ImmunoResearch	anti-IgA	109-005-011
21	Jackson ImmunoResearch	anti-IgM	109-005-129
22	Pierce	BSA	23225
Detection Ab	Jackson ImmunoResearch	anti-human IgG-PE	109-115-098

Table S2. Agreement between RBD IgG ELISA and saliva multiplex assay

	**Saliva**	**RBD**	**PPA**
All RBD+	15	39	38.5
RBD+, high NR	13	22	59.1

	**Saliva**	**RBD**	**NPA**
All RBD-	37	40	92.5
RBD-, low NR	26	27	96.3
